# Unveiling the synaptic selectivity of tau oligomers: implications for targeted therapeutics in tauopathies

**DOI:** 10.1002/alz70855_103322

**Published:** 2025-12-23

**Authors:** Shrinath Kadamangudi, Laura Sanchez, Agenor Limon, Rakez Kayed, Giulio Taglialatela

**Affiliations:** ^1^ University of Texas Medical Branch, Galveston, TX, USA

## Abstract

**Background:**

Synaptic dysfunction is a hallmark of tauopathies and a key driver of cognitive decline. Tau pathology progression correlates with cognitive deterioration, underscoring the therapeutic potential of targeting tau at the synapse. However, cellular and regional susceptibilities to tau pathology complicate therapeutic efforts, and the mechanisms underlying synaptic vulnerability in human tauopathies remain poorly understood.

**Methods:**

Post‐mortem brain tissue from control and primary age‐related tauopathy (PART) cases was used to investigate synaptic vulnerability to soluble tau oligomers (tauO), highly toxic pathological conformers. Definite PART samples, characterized by selective tau accumulation without co‐pathologies (e.g., Aβ, α‐syn, TDP‐43), were studied. Synaptosomes from hippocampus and temporal cortex were: (1) characterized for tau pathology (*p*‐tau, tauO, total tau) via western blotting, (2) treated with recombinant tau oligomers (r‐tauO) and analyzed by flow cytometry (FC) immunophenotyping to examine pre‐/post‐synaptic and GABAergic/glutamatergic tauO interactions, (3) microtransplanted into frog oocytes for two‐electrode voltage clamp (TEVC) recordings of GABA and AMPA currents, and (4) examined via EM immunogold to confirm tauO‐synapse interactions. Additionally, brain‐derived tau oligomers (BDTO) from PBS‐soluble PART hippocampal lysates were isolated via co‐immunoprecipitation and analyzed by LC‐MS/MS to identify synaptic BDTO interactomes.

**Results:**

Outcome‐1 | In control synaptosomes treated with r‐tauO, FC and EM revealed that tauO preferentially engage pre‐synaptic terminals and associate with synaptic vesicles. TauO showed higher affinity for GABAergic synapses (FC) and selectively potentiated GABAergic currents (TEVC). Outcome‐2 | In PART synaptosomes, tau aggregates (tauO and PHF‐tau) were significantly elevated in the hippocampus compared to the temporal cortex (western blot), correlating with a decreased excitatory/inhibitory (E/I) ratio, suggesting a pro‐inhibitory shift in regions with elevated tau pathology. Outcome‐3 | PART BDTO interactomes revealed significant enrichment of pre‐synaptic proteins involved in synaptic vesicle cycling, aligning with Outcome‐1.

**Conclusions:**

This study provides the first direct evidence of selective synaptic vulnerability to tauO in human brain tissue, revealing a preference for pre‐synaptic and inhibitory synapses. These findings challenge post‐synaptic‐focused therapeutic strategies, such as targeting NMDA and acetylcholine pathways. By identifying pre‐synaptic vesicle cycling as a tauO target, our study underscores the need for tau therapeutics tailored to specific synaptic populations and circuits.

